# Label-Free Electrochemical Immunosensor Based on Conjugated Polymer Film Coated Disposable Electrode for Ultrasensitive Determination of Resistin Potential Obesity Biomarker

**DOI:** 10.1021/acsabm.3c01231

**Published:** 2024-02-23

**Authors:** Elif Burcu Aydın, Muhammet Aydın, Mustafa Kemal Sezgintürk

**Affiliations:** † Scientific and Technological Research Center, Tekirdaǧ Namık Kemal University, Tekirdaǧ, Turkey 59030; ‡ Bioengineering Department, Faculty of Engineering, Çanakkale Onsekiz Mart University, Çanakkale, Turkey 17100

**Keywords:** resistin, obesity biomarker, conducting thiophene polymer, disposable biosensor

## Abstract

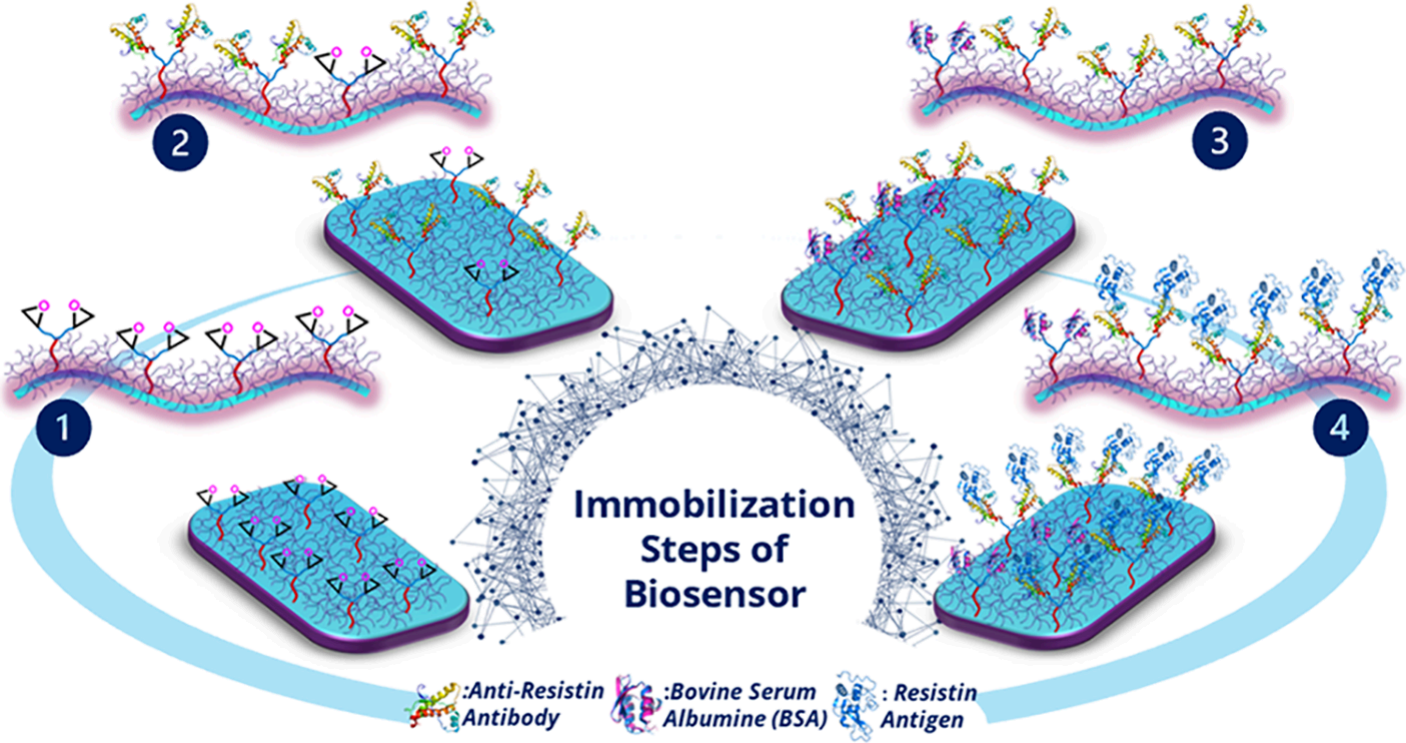

A new label-free immunosensor was designed for sensitive detection of resistin obesity biomarker in human biological fluids. To construct a sensing interface, the monomer of double epoxy groups-substituted thiophene (*TdiEpx*) was synthesized for the fabrication of the biosensing system. A disposable indium tin oxide sheet was first modified by electrochemical polymerization of the *TdiEpx* monomer, and this robust and novel surface was characterized using different spectroscopic and electrochemical analyses. The double epoxy ends were linked to the amino ends of anti-resistin, and they served as binding points for the covalent binding of biomolecules. The double epoxy ends present in each *TdiEpx* monomer ensured an extensive surface area, which improved the quantity of attached anti-resistin. The determination of resistin antigen was based on the specific coupling of resistin with anti-resistin, and this interaction hindered the electron transfer reaction. The immunosensor introduced a wide linear range of 0.0125–15 pg/mL, a low detection limit of 4.17 fg/mL, and an excellent sensitivity of 1.38 kohm pg mL^–1^ cm^2^. In this study, a sandwich enzyme-linked immunosorbent assay spectrophotometric method was utilized as a reference technique for the quantitative analysis of resistin in human serum and saliva samples. Both measurements in clinical samples displayed correlations and high-correlation coefficients. In addition, this immunosensor had good storage stability, acceptable repeatability and reproducibility, high specificity, and good accuracy. The proposed immunosensor provided a simple and versatile impedimetric immunosensing platform and a promisingly sensitive way for clinical applications.

## 1 Introduction

The World Health Organization (WHO) describes obesity as “a condition of abnormal or excessive fat accumulation in adipose tissue, to the extent that health may be impaired”. The relevance of obesity has sharply increased in the past few years, and it is now considered a major health problem worldwide.^1^ According to a notice issued by the WHO in 2016, over 1.9 billion adults (persons over the age of 18) were overweight, and more than 600 million individuals were obese. It is supposed that by 2030, 2.16 billion individuals will be overweight and 1.12 billion people will be obese.^2^ The increasing number of obese people in industrialized regions is chiefly associated with modern lifestyle factors such as nutrition and leisure activities.^3,4^ Obesity is an essential risk determinant for cardiovascular diseases, hypertension, type 2 diabetes, and several cancers such as colorectal, renal cell, breast, esophageal, pancreatic, and liver.^5−7^

Resistin is a cysteine-rich polypeptide, and there is a potential connection between obesity and type 2 diabetes. Resistin is also known as an adipose tissue-specific secretary factor, which is encoded by the RETN gene present on chromosome 19.^5,8^ Resistin, also known as adipocyte-secrete-factor, is a new adipocytokine produced from adipocytes and monocytes and is involved in inflammatory processes such as atherosclerosis, rheumatic diseases, and malignancies.^9,10^ The serum resistin concentrations in obese and lean volunteers were reported as 24.58 ± 12.93 ng/mL (*n* = 64) and 12.83 ± 8.30 ng/mL (*n* = 15), respectively.^11^ In order to determine human resistin levels, enzyme-linked immunosorbent assays are primarily utilized, but they are relatively time-consuming and high-cost.^12,13^ Electrochemical biosensors have received widespread attention due to their simple instrumentation, fast response time, and inexpensive cost. Typically, antibody-based electrochemical biosensors are useful tools for the determination of biological markers.^14^ The generated bio-recognition events cause changes in the interfacial properties of the electrode, and the related changes can be measured with the EIS technique without requiring a label.^15−17^

The key constituent of any biosensing system is the recognition element.^18−20^ Electrochemical biosensors and sensors can be fabricated by electropolymerizing of monomers or coating of the electrode surface with chemically synthesized polymers.^21^ Conjugated polymers are a significant class of functional substances that have usually been utilized to construct electrochemical systems due to their attractive and adjustable chemical, electrical, and constructive features.^22,23^ Polythiophene is one of the most important π-conjugated polymers for biosensors, supercapacitors, electrochromic devices, and thermal conductors because of its good electrical conductivity, mechanical robustness, and environmental stability.^24^ A few methods, such as chemical oxidation, electrochemical oxidation polymerization, and oxidative chemical vapor deposition, have been employed to prepare the polymeric thin films by using thiophene monomers. The most popular method to generate a polythiophene polymer layer is electrochemical oxidation polymerization, which provides simple control of the polymerization degree through tuning of the applied potential.^25,26^ Shoja et al. (2017) developed a voltammetric bi-enzyme biosensing system for dopamine. They electropolymerized thiophene monomer to immobilize the amino acid-*d* oxidase (DAAO) and hemoglobin proteins.^27^ Uygun et al. (2009) prepared a polythiophene/SiO_2_ nanocomposite for glucose oxidase enzyme immobilization for glucose sensing.^28^ Fusco et al. (2017) fabricated a glucose-sensing enzyme biosensor by modifying the working electrode with a polytiophene polymer/thiol functionalized multiwalled carbon nanotube/glucose dehydrogenase enzyme.^29^ As mentioned above, commercially available polythiophene polymers have been used in the literature for electrode modification. In this study, a thiophene monomer carrying double epoxy groups (*TdiEpx*) was synthesized, and these epoxy terminals were utilized for the binding of target resistin antibodies. In this context, there was no need for any other material for the binding of antibodies. In addition, the excellent and controllable electrochemical properties of *TdiEpx* ensured a suitable supporting matrix for the binding of anti-resistin molecules. With their unique electron transfer properties and desirable groups present on the polymer backbone, *TdiEpx* is a good matrix for surface modifications. Furthermore, the P(*TdiEpx)* polymer was a new matrix for anti-resistin immobilization.

**Scheme 1:**
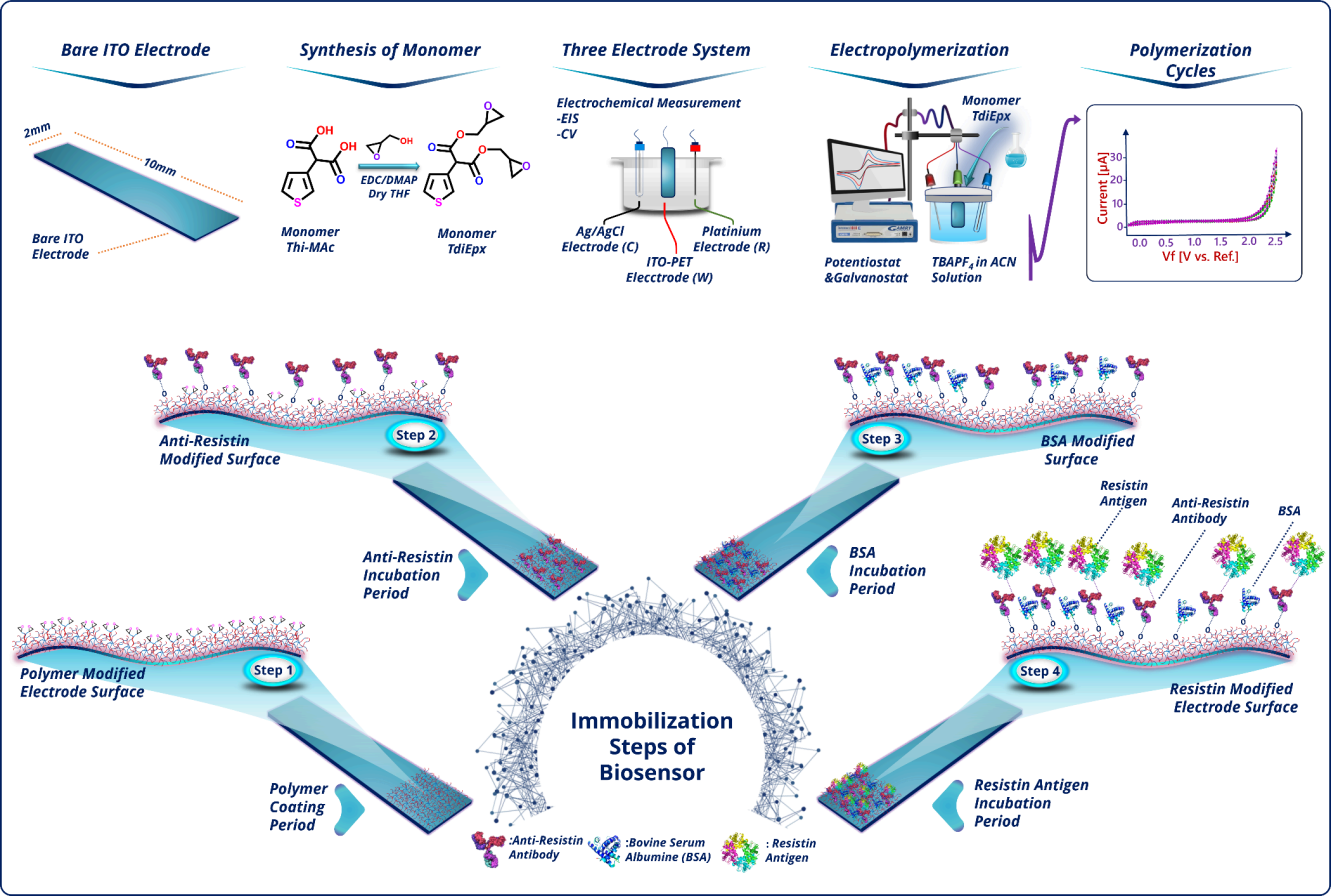
Fabrication Stages of the Resistin Electrochemical Biosensor

Herein, a biosensing platform based on electropolymerized *TdiEpx* polymer film was employed for the detection of resistin biomarker. The controllable electrochemical procedure for the fabrication of the immunosensor provided a polymeric thin film formation on the ITO electrode. Because of the epoxy groups on the polymer backbone, suitable attachment of the antibodies was achieved. Additionally, the *P(TdiEpx)* conjugated polymer offered an advanced surface for rapid electron transfer, which significantly enhanced the response of the prepared surface. This modification provided a stable sensing interface with outstanding stability and sensitivity. The electrode surface after each stage of the fabrication protocol (Scheme 1) was characterized by EIS, cyclic voltammetry (CV), scanning electron microscopy (SEM), atomic force microscopy (AFM), and a Fourier transform infrared spectrometer (FTIR). The parameters that affect biosensor response were optimized by studying the electropolymerization cycles of *TdiEpx* monomer, immobilization concentrations of anti-resistin, and incubation times of anti-resistin and resistin. The suggested immunosensor had a wide linear range from 0.0125 to 15 pg/mL with a correlation coefficient of *R*^2^ = 0.9987 and was successfully applied for the measurement of resistin in human samples (serum and saliva) with satisfactory analysis results. The suggested biosensor also has great potential for the analysis of different target biomarkers.

## 2 Experimental Section

### 2.1 Materials and Chemicals

Tetrabutylammonium tetrafluoroborate (TBAPF_4_), sodium perchlorate/lithium perchlorate (NaClO_4_/LiClO_4_), and tetrabutylammonium hexafluorophosphate (TBAPF_6_) were from Sigma-Aldrich. Anti-resistin antibody (0.75 mg/mL), resistin from mouse (25 ug), neuron-specific enolase human (NSE, 10 μg), vascular endothelial growth factor (VEGF), calreticulin human (CALR, 10 μg), p53 protein human (50 μg), GM2-activator protein (GM2A, 3.70 mg/mL), human serum, and bovine serum albumin (BSA) were from Sigma-Aldrich. Elabscience Biotechnology Inc. (USA) provided the ELISA Resistin kit.

### 2.2 Apparatus

The electrochemical tests (EIS, CV, and single frequency impedance (SFI)) were carried out on the Gamry Reference 1000 with a three-electrode system. Indium tin oxide-coated polyethylene terephthalate film (ITO), platinum wire, and Ag/AgCl were utilized as a working, counter, and reference electrodes, respectively. A Thermo-Orion 3-star was utilized for pH measurements. Polymer film characterization and immobilization of anti-resistin were confirmed with FTIR (Bruker Vertex 70). The surface morphologies of electrodes were analyzed by SEM. SEM measurements were conducted on the FEG-250 at an accelerating voltage of 50 kV. Energy-dispersive X-ray spectroscopy (EDX) was done on FEG-250 EDAX, and the utilized acceleration voltage and spot size were 30 kV and 6.0, respectively. Furthermore, the surface morphology of modified electrodes was also analyzed with AFM (Nanomagnetics, Turkey). The analysis used a scan speed of 2 μm s^–1^ with a resolution of 256 pixels per line and was conducted in tapping mode.

### 2.3 Synthesis of Monomer (*TdiEpx*)

The *TdiEpx* monomer, a colorless liquid, was synthesized with the use of the Steglich esterification process between 3-thiophenemalonic acid and glycidol under nitrogen gas (yield, %58, 0.42 g). FTIR (ATR; cm^–1^): 3103; 2921; 2852; 1733 (C=O); 1412; 1254; 1137; 1080; 1007; 908; 844; 761; 685; 612; 490. Raman (λ_laser=780 nm_): 3109; 3006; 2932; 1738 (C=O); 1412; 1258; 1153; 1084; 992; 947; 925; 860; 836; 763; 677; 534; 461. ^1^H NMR (400 MHz, CDCl_3_, ppm): 7.33 (H_a_, 1H), 7.01 (H_b_, 1H), 7.16 (H_c_, 1H), 5.08 (H_d_, 1H), 4.44 (H_e1_, 1H), 3.96 (H_e2_, 1H), 3.20 (H_f_, 1H), 2.83 (H_g1_, 1H) ve 2.63 ppm (H_g2_, 1H).

### 2.4 Preparation of the Electrochemical Biosensor

The electrodes were ultrasonically cleaned with acetone, soap solution, and ultrapure water for 10 min. Subsequently, they were dried under argon gas at room temperature. Electropolymerization of *TdiEpx* monomer was performed via CV on a clean ITO in acetonitrile including TBAPF_6_ (0.1 M) at a cycling range from −0.2 to 2.5 V at a scanning rate of 50 mV/s. Then, the prepared electrodes were dipped in an anti-resistin solution for 45 min and rinsed with water to eliminate unspecific physical adsorption. Following incubation, the anti-resistin-modified electrodes were incubated in BSA for 60 min to block residual active sites present on the ITO/*P(TdiEpx)*/anti-resistin electrode. Finally, the prepared electrode was ready for detection of resistin biomarker, and it was dipped in the antigen solution for 45 min. If the electrode was not utilized for resistin biomarker analysis, it could be stored at 4 °C for the next applications.

### 2.5 Electrochemical Measurements

The stepwise assembly of the proposed electrochemical biosensor was detected through EIS and CV analyses in a ferri–ferro redox probe solution (5 mM, pH 7.4). CV and EIS analyses illustrated current responses and resistances linked to the interfacial electron transfer with immobilized biomolecules on the electrode surface. EIS analyses were performed in the presence of a 5 mM [Fe(CN)_6_]^3-/4–^ redox couple prepared in 0.1 M KCl, and the EIS frequency range was from 0.05 to 50000 Hz at a DC potential of 0 V. For the electropolymerization process, CV measurements were conducted from −0.2 to 2.5 V at a scan rate of 0.05 V/s. SFI represented the variations in impedance at a 30 Hz monitoring frequency during the anti-resistin–resistin immunocomplex formation.

### 2.6 Resistin Quantification Principle

For quantification of the resistin biomarker, the BSA attached electrodes were reacted with different concentrations of resistin for 45 min, and subsequently, the prepared electrodes were washed with ultrapure water. After incubation in a resistin solution, an immunocomplex formed between the anti-resistin and resistin, resulting in a protein barrier formation on the working electrode surface. The impedimetric responses increased with the increments in resistin concentrations. The increases in the EIS signals were proportional to the increasing concentrations of resistin, and this relationship was used for the measurement of target antigen.

### 2.7 Serum and Saliva Sample Analyses

The resistin levels present in serum and saliva samples were examined with the proposed immunosensor and ELISA kit. Before the analyses, serum and saliva samples were diluted 20- and 50-fold with phosphate buffer for biosensor and ELISA measurements, respectively. In this study, ELISA analysis was utilized as a reference method to determine the reliability of the assay. To further research the suitability of the developed immunosensor in serum and saliva, resistin was added at concentrations of 1 and 7.5 pg/mL to serum and saliva samples, and the prepared samples were studied according to the method described above.

## 3 Results and Discussion

The *TdiEpx* monomer synthesis pathway and construction process of the suggested biosensor are presented in Scheme 1. As seen in the fabrication scheme, first of all, the *TdiEpx* monomer was electropolymerized on the ITO surface (Step 1). Later, monoclonal anti-resistin were covalently attached onto the modified surface (Step 2). To further block the nonspecific binding site on electrodes, BSA molecules were utilized (Step 3). Finally, the anti-resistin-linked electrodes were incubated with resistin for 45 min to achieve specific interaction (Step 4).

### 3.1 Characterization of the Impedimetric Resistin Biosensor

#### 3.1.1 FTIR and EDX Characterization of the Different Modified Electrode Surfaces

The Steglich esterification method was used to synthesize the double epoxy functional group-substituted thiophene monomer (*TdiEpx*). Scheme 1 lists the *TdiEpx* chemical structure and preparation process. To demonstrate the effectiveness of the monomer synthesis method, the chemical structure of the monomer was studied using various spectral techniques (FTIR, Raman, mass, and proton NMR spectroscopy). The supplementary data file included a detailed presentation of the results.

**Figure 1:**
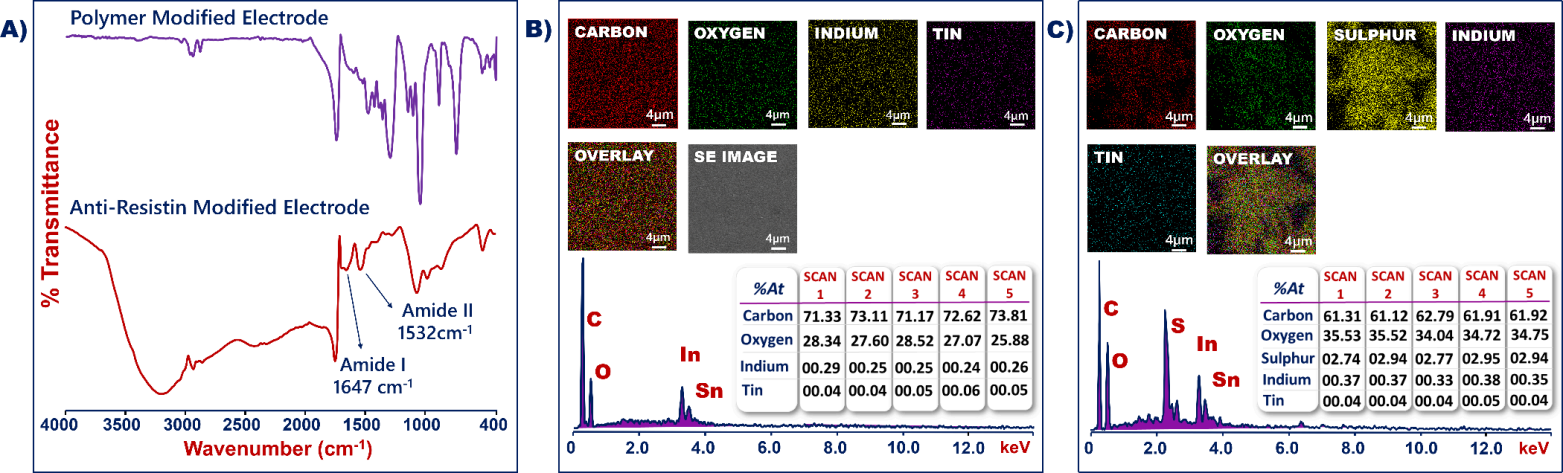
FTIR (A) and SEM-EDX (B and C) analyses results.

*P(TdiEpx)* polymer electropolymerization and anti-resistin antibody attachment onto the electrode surface were confirmed using FTIR characterizations. The FTIR spectrum of ITO/*P(TdiEpx)* is shown in Figure 1A (purple line), and the peaks at 3061, 1241, 965, and 836 cm^–1^ belonged to the C–H in the oxirane ring, symmetric ring, and asymmetric and symmetric C–O–C stretching vibrations, respectively. Furthermore, the sharp peak at 1715 cm^–1^ illustrated the stretching vibration of carbonyl groups. The peak at 1511 cm^–1^ was assigned to the C=C stretching in thiophene rings. Two peaks at 871 and 722 cm^–1^ displayed the C–S stretching of the thiophene ring. The results presented above demonstrated the successful formation of a *P(TdiEpx)* on the ITO surface. Figure 1A (red line) illustrates the FTIR spectrum of ITO/*P(TdiEpx)*/anti-resistin, and broad and intense bands were obtained at ∼1647 cm^–1^ (amide I) and at ∼1532 cm^–1^ (amide II), respectively. These bands illustrated the successful immobilization of anti-resistin molecules.

The typical EDX patterns of bare and *P(TdiEpx)* functionalized ITO electrodes, along with SEM images of the corresponding electrode surface areas, are shown in Figure 1B,C, respectively. EDX measurements were performed 5 times in several points. The spectra showed the presence of S (from the thiophene ring of *P(TdiEpx)*) and other elements on the ITO surface. The S element was the main difference between the bare and *P(TdiEpx)* polymer-coated electrodes, as shown in Figure 1B,C. In addition, Figure 1C illustrates that the S element was homogeneously distributed over the modified electrode surface area.

#### 3.1.2 Electrochemical Behaviors of the Fabricated Biosensor Surfaces

The electrochemical behaviors of each stage of the current resistin immunosensor were investigated using EIS and CV techniques. EIS spectra are recorded to follow the performance of this system throughout biosensor construction. The semicircle part of the Nyquist curve at higher frequencies is associated with electron transfer-limited reactions, and it illustrates the changes at each modification step. Besides, the semicircle diameter is associated with the charge transfer resistance (*R*_ct_).^29,30^ In addition, the EIS spectra are fitted using the Randles’ equivalent circuit, which includes four components: (1) the electrolyte solution ohmic resistance, *R*_s_; (2) the Warburg impedance, *W*; (3) the constant phase element, CPE; and (4) *R*_ct_. In general, *R*_ct_ is an indicator of the electrostatic barrier and reaction kinetics present at the electrode–electrolyte interface.^31−33^

#### 3.1.3 SEM and AFM Characterization

**Figure 3:**
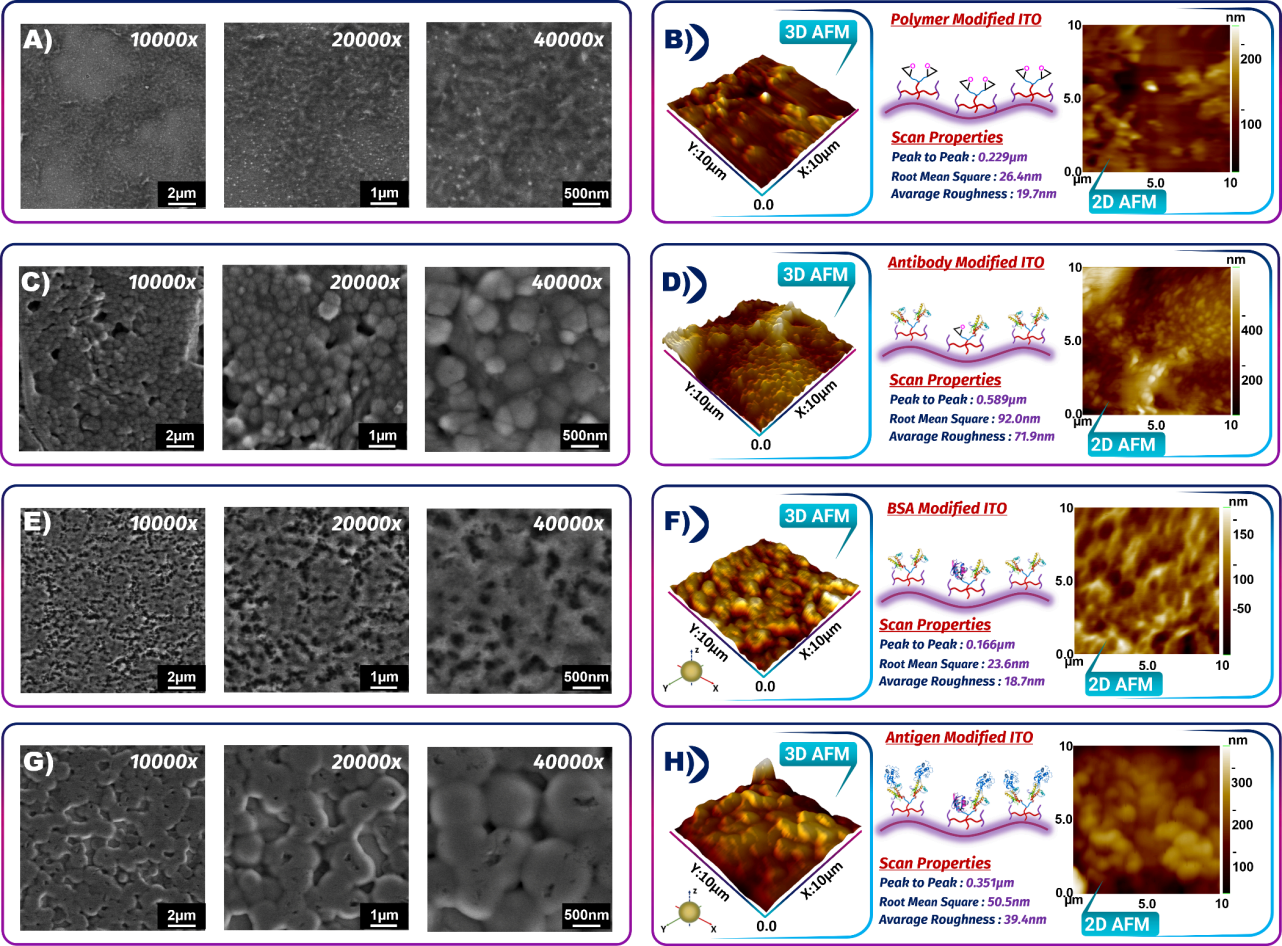
SEM and AFM images of each step to produce the resistin biosensor.

In order to characterize the impact of the modification procedure on the morphologies of the ITO electrode surface, SEM and AFM were utilized to characterize the electrode surface during each modification. Panels A and B of Figure 3 illustrate the SEM and AFM images of the clean ITO surface, respectively, and the surface of the electrode was relatively smooth. The average roughness (*R*_a_) of the clean electrode was 0.90 nm (Figure 3B). The morphology of the bare ITO electrode was remarkably changed by electrochemical polymerization of the *P(TdiEpx)* layer (Figure 3C). Figure 3C indicates the successful coating of the *P(TdiEpx)* film on the ITO electrode. The *P(TdiEpx)* polymer film formation increased *R*_a_ (19.70 nm, Figure 3D). After binding anti-resistin on the electrode surface, its morphology changed. As can be observed in Figure 3E, the changes on the surface image proved the successful attachment of bio-recognition elements. The SEM image of this surface was supported by the AFM image of the ITO/*P(TdiEpx)*/anti-resistin electrode, and the *R*_a_ was measured as 71.9 nm (Figure 3F). After the BSA blocking stage, the electrode morphology was completely different, and BSA immobilization caused a layer on the ITO (Figure 3H). The *R*_a_ of this surface was 18.7 nm, and a relatively smooth surface was observed (Figure 3I). The specific interaction between anti-resistin and its target formed a proteinous layer and changed the surface image of the electrode. An increase in *R*_a_ (39.4 nm) indicated that the introduction of resistin resulted in a protein biolayer on the functionalized electrode surface (Figure 3J). The SEM and AFM characterizations provide further confirmations of the EIS and CV observations by showing changes in the surface topography of the electrode after anti-resistin immobilization and resistin target antigen capture.

### 3.2 Optimization Conditions of Analytical Resistin Biosensor

**Figure 4:**
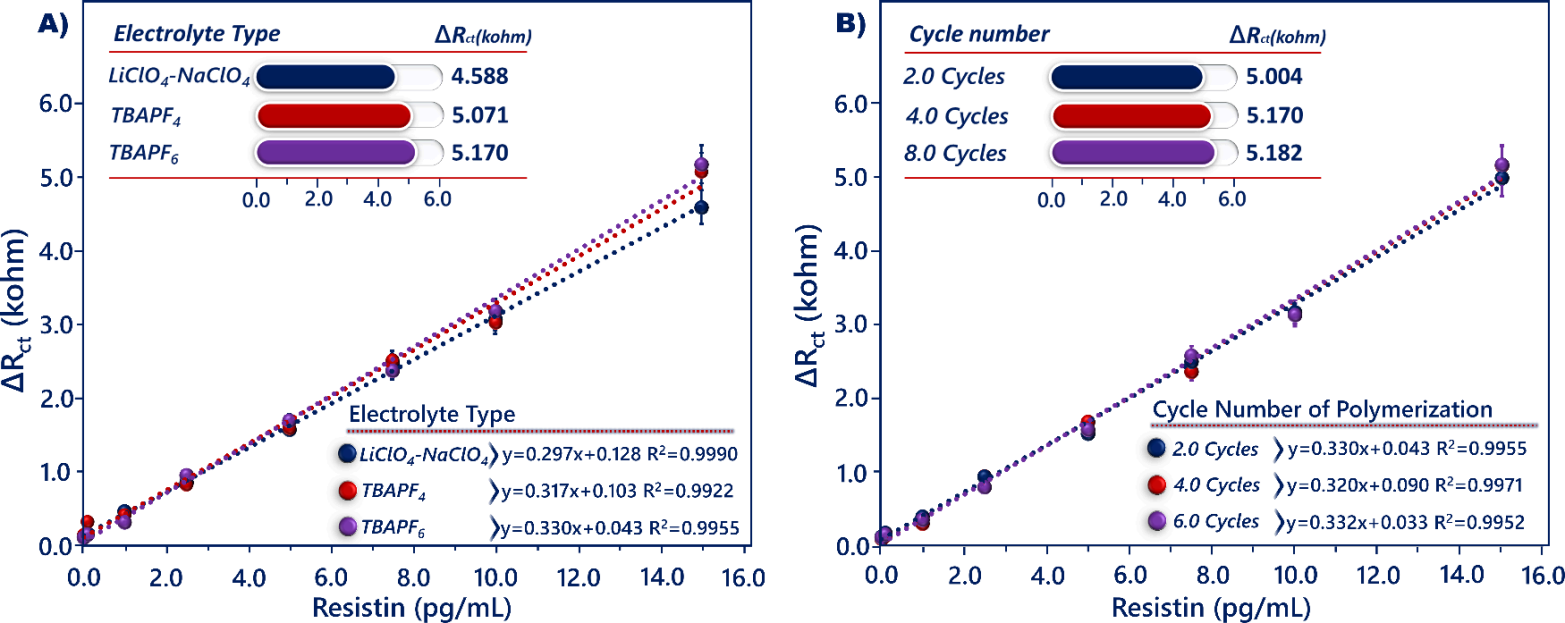
Optimization of the utilized electrolyte type (A) and number of electropolymerization cycles (B).

To measure an optimum electrochemical signal, effective parameters such as electrolyte type, number of electropolymerization cycles, anti-resistin concentration, and incubation times of anti-resistin and resistin were optimized. First, the type of electrolyte was selected. Different electrolyte solutions, such as TBAPF_4_, TBAPF_6_, and LiClO_4_/NaClO_4_ were prepared. The maximum impedimetric signal was obtained with the use of a TBAPF_6_ electrolyte solution (Figure 4A).

The number of electropolymerization cycles was important for the thickness of the polymer layer, and a stable polymer layer could be obtained after the application of the optimum number of electropolymerization cycles. An excessively thick layer might impede electron transmission between the electrode surface and electrode solution, resulting in a high *R*_ct_ value. Conversely, an excessively thin layer would prevent sufficient biomolecules from attaching to the electrode surface, which would result in a low biosensor response. The increase in cycle number increased the number of epoxy ends present on the ITO electrode and caused a thicker polymer layer. As seen in Figure 4B, it is obvious that the *R*_ct_ values increased from 2 to 8 cycles, and therefore, 4 cycles were chosen as the optimal number of electropolymerization cycles.

**Figure 5:**
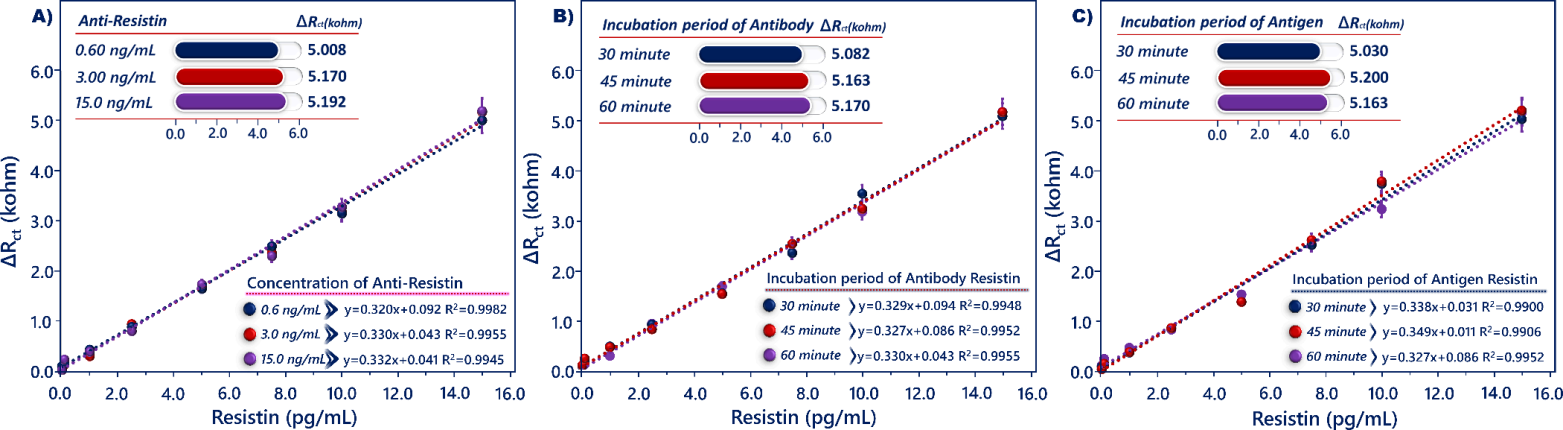
Optimization of anti-resistin concentration (A), anti-resistin (B), and resistin incubation times (C).

To investigate the influence of anti-resistin concentration, different amounts of anti-resistin were utilized to obtain a more sensitive biosensor. The Δ*R*_ct_ value increased with the increasing anti-resistin concentrations (0.6, 3, and 15 ng/mL), and the immunosensor responses of 3 ng/mL utilizing the biosensor and 15 ng/mL utilizing the biosensor were similar. Therefore, 3 ng/mL anti-resistin was the most appropriate concentration due to the cost of the immunosensor (Figure 5A). The amount of bound antibody on the ITO surface and the amount of connected antigen with its antibody on the electrode surface varied with incubation time. Because of this, the incubation times of anti-resistin and resistin were optimized. Based on the obtained results, the impedimetric response obtained after a 30 min incubation was low because enough anti-resistin molecules did not immobilize on the ITO surface in 30 min. With increasing the incubation duration of anti-resistin, Δ*R*_ct_ increased, and 45 min was chosen as the optimum immobilization duration for anti-resistin (Figure 5B). Last, the incubation time of resistin was optimized. As seen in Figure 5C, short incubation time caused a low signal because effective binding of the resistin did not perform, and this time was too short for binding of the sufficient amount of resistin. With the increase in incubation time of resistin, Δ*R*_ct_ increased, and 45 min was decided as the optimum immobilization time for resistin.

### 3.3 Performance of the Resistin Biosensing System

**Figure 6:**
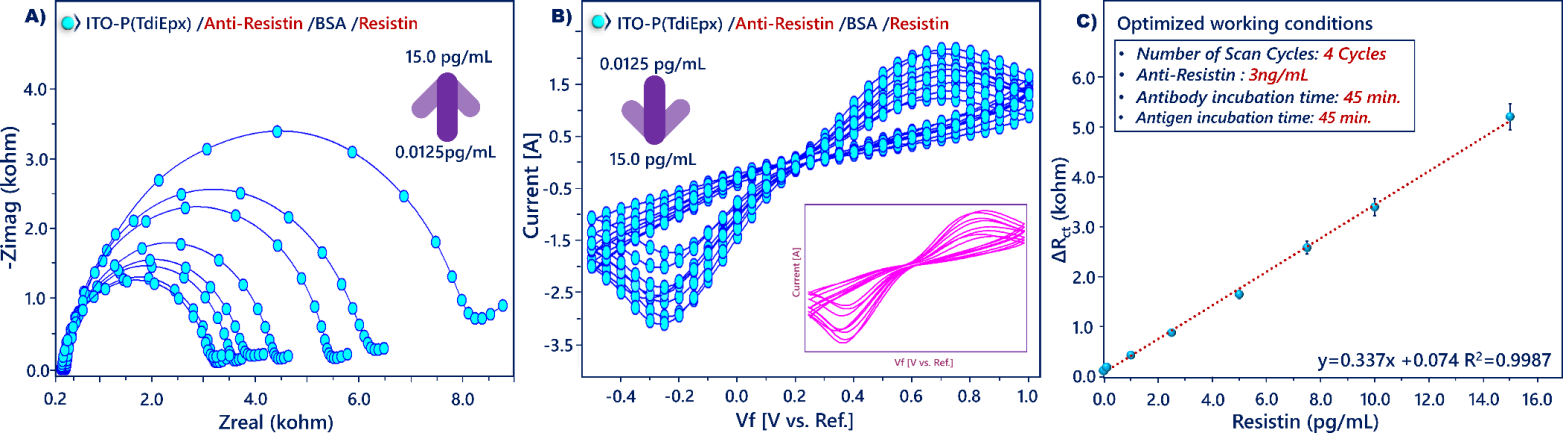
EIS (A) and CV (B) responses and calibration curve (C) of the proposed biosensor.

The analytical performance of the constructed biosensor was evaluated by the analysis of a different concentrations of resistin by using EIS technique. It could be seen in Figure 6A that the EIS signals increased along with the increase in resistin levels because resistin antibodies specifically captured the resistin antigens, and thus, this interaction prevented the interfacial electron transfer significantly (Table S1B). In the same way, the increase in resistin antigens captured by anti-resistin antibodies resulted in decreases in CV currents (Figure 6B). The calibration plot of the system was drawn using the changes in Δ*R*_ct_, which was the difference between *R*_ct_(target resistin) and *R*_ct_(BSA). The Δ*R*_ct_ values were computed by a curve fitting technique, wherein the electrochemical system was modeled as an electrical circuit. The calibration curve was obtained by plotting Δ*R*_ct_ versus resistin concentration, and a great linear relationship with the resistin concentration from 0.0125 to 15 pg/mL was obtained (Figure 6C). The response of the biosensor to antigen concentrations higher than 15 pg/mL is given in Figure S4. The calibration curve was linear, and the calibration regression equation was Δ*R*_ct_ = 0.337[resistin (pg/mL)] + 0.074, *R*^2^ = 0.9987. The LOD (3*s*/*m*), limit of determination (LOQ, 10*s*/*m*) and sensitivity were 4.17 fg/mL, 13.9 fg/mL, and 1.378 kohm pg mL^–1^ cm^2^, respectively (*s* = blank standard deviation; *m* = slope of calibration curve). The proposed immunosensor illustrated an effective surface design to obtain a wide linear range. The LOD of this method (4.17 fg/mL) was lower by more than 400 times than the ELISA method for quantification of resistin biomarker Table 1A. Aside from this advantage, the convenient fabrication process and simplicity of the method make it a suitable system for resistin quantification. Furthermore, the high performance of the biosensor can account for the favorable orientation of bio-recognition elements in the biosensor design. It also means that the anti-resistin immobilized electrode can recognize the target analyte since the *P(TdiEpx)* polymer creates an outstanding platform for anti-resistin antibody immobilization. In addition, through the functional epoxy groups of conducting polymer, strong amide bond formation between antibodies and *P(TdiEpx)* polymer was achieved. Thus, the anti-resistin antibody-modified electrode provided fast and selective determination of resistin antigen.

**Table 1:** Comparison of Resistin Analysis Techniques (A) and Detection of Resistin in Serum (B) and Saliva (C) Samples Using the Suggested Sensor

(A) Analysis techniques			
Detection method	Linear detection range (pg/mL)	LOD (fg/mL)	Ref
ELISA	625 to 2 × 10^4^	10^5^	MyBiosource
ELISA	2–400	2000	Ray BioTech
ELISA	78.1–5000	24000	Abcam
ELISA	31.25–2000	18750	Elabscience
*P(TdiEpx)* modified biosensor	0.0125–15	4.17	This work

(B) Serum Samples						
Sample	Measd (pg/mL)	Added (pg/mL)	Total (pg/mL)	SD and CV (%)^a^	Recovery (%)	Rel diff (%)
1	6.66	1.00	7.61	0.17–2.17	99.34	–0.66
1	6.57	1.00	7.85		103.64	3.64
1	6.66	7.50	14.14	0.19–1.32	99.84	–0.16
1	6.57	7.50	14.41		102.37	2.37
2	3.99	1.00	4.88	0.04–0.85	97.80	–2.20
2	4.08	1.00	4.94		97.26	–2.74
2	3.99	7.50	11.56	0.02–0.18	100.58	0.58
2	4.08	7.50	11.53		99.55	–0.45
3	1.48	1.00	2.63	0.02–0.79	105.86	5.86
3	1.50	1.00	2.66		106.29	6.29
3	1.48	7.50	9.19	0.04–0.46	102.26	2.26
3	1.50	7.50	9.13		101.40	1.40
4	4.56	1.00	5.46	0.05–0.92	98.18	–1.82
4	4.59	1.00	5.53		98.94	–1.06
4	4.56	7.50	12.03	0.03–0.26	99.81	–0.19
4	4.59	7.50	12.08		99.94	–0.06
5	11.23	1.00	12.65	0.09–0.71	103.45	3.45
5	11.11	1.00	12.78		105.46	5.46
5	11.23	7.50	18.71	0.02–0.11	100.68	0.68
5	11.11	7.50	18.74		100.68	0.68

(C) Saliva Samples						
Sample	Measd (pg/mL)	Added (pg/mL)	Total (pg/mL)	SD and CV (%)^a^	Recovery (%)	Rel diff (%)
1	9.25	1.00	10.28	0.23–2.28	100.38	0.38
1	9.16	1.00	9.96		98.04	–1.96
1	9.25	7.50	16.60	0.02–0.13	99.16	–0.84
1	9.16	7.50	16.57		99.51	–0.49
2	3.04	1.00	4.02	0.08–2.06	99.49	–0.51
2	3.19	1.00	4.14		98.80	–1.20
2	3.04	7.50	10.49	0.13–1.19	99.51	–0.49
2	3.19	7.50	10.67		99.79	–0.21
3	1.51	1.00	2.69	0.17–6.53	106.97	6.97
3	1.44	1.00	2.45		100.36	0.36
3	1.51	7.50	9.07	0.18–1.97	100.61	0.61
3	1.44	7.50	8.82		98.62	–1.38
4	1.65	1.00	2.69	0.13–4.84	101.46	1.46
4	1.62	1.00	2.51		95.81	–4.19
4	1.65	7.50	9.10	0.21–2.34	99.43	–0.57
4	1.62	7.50	8.80		96.50	–3.50
5	1.03	1.00	2.09	0.02–1.01	103.37	3.37
5	1.00	1.00	2.06		103.42	3.42
5	1.03	7.50	8.45	0.18–2.15	99.11	–0.89
5	1.00	7.50	8.71		102.53	2.53

a SD, standard deviation; CV, coefficient variation.

**Figure 7:**
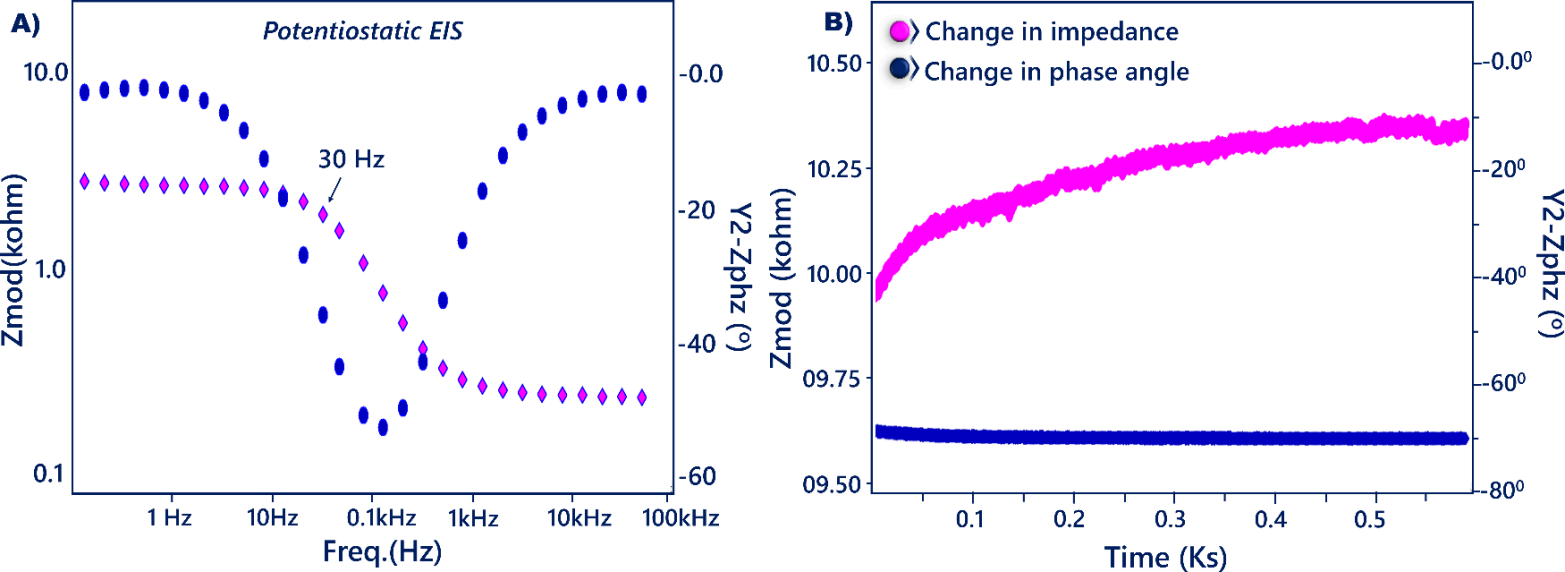
Bode curve (A) and SFI analysis of resistin (B).

SFI is an impedance analysis process where a constant frequency is utilized as an induced signal in place of a wide frequency range. This method reduces the complication in signal attainment and processing, and thus a favorable, easy, and low-cost analysis is performed.^34^ In this analysis, impedance was recorded at a constant frequency obtained by Bode plot, and impedance changes were monitored in phosphate buffer containing target antigen.^35^ In this study, 30 Hz (Figure 7A) was used as the measurement frequency and the proposed immunosensor was immersed in a resistin solution. As seen in Figure 7B, increases were measured in impedance. This change illustrated the specificity of the proposed biosensor to target protein. The SFI analysis provided a sensitive way to monitor the electrical change occurring at the bioelectrode.

**Figure 8:**
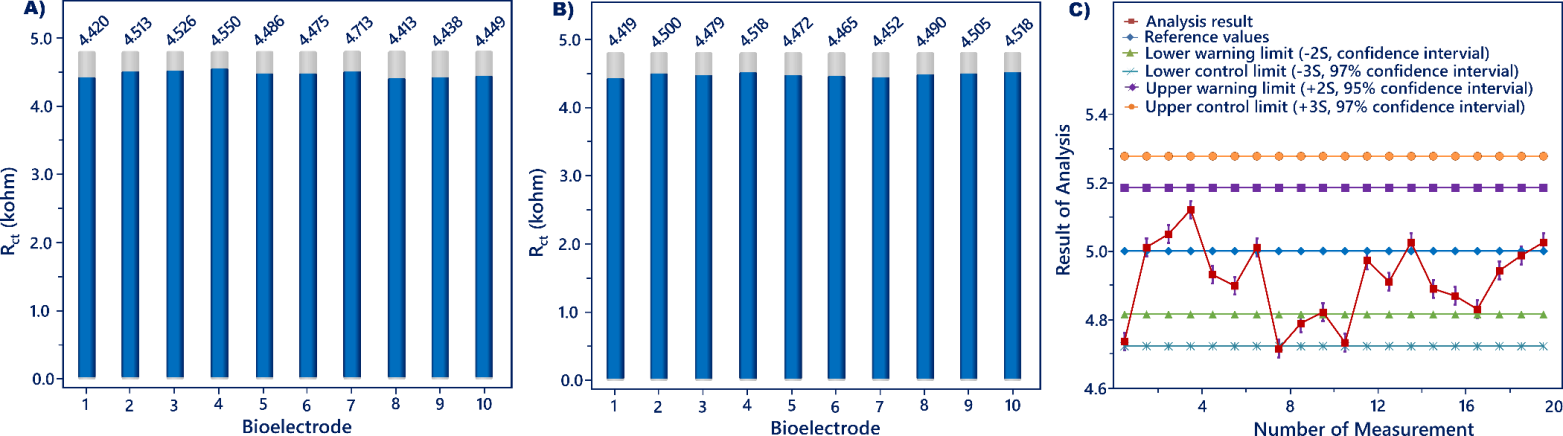
Biosensor repeatability (A), reproducibility (B), and quality control curve (C).

The repeatability of the developed sensor was analyzed by measuring resistin concentrations (0.1, 5, and 10 pg/mL) with 30 bioelectrodes prepared under the same status, and the analysis result for 5 pg/mL is illustrated in Figure 8A. The repeatability data were statistically analyzed with Grubbs’ and Dixon’s outlier tests. The relative standard deviations (RSD) of repeatability test results and *p* values of the Grubbs’ and Dixon’s tests are summarized in Table S2A. The *p* values were lower than critical values, and accordingly, the outliers were not present in the analysis results.

Apart from the repeatability test, the reproducibility test was also performed. The reproducibility of the resistin biosensor was measured by using 30 electrodes constructed under the same test conditions, and these electrodes were utilized for resistin analysis (0.1, 5, and 10 pg/mL). The analysis result to 5 pg/mL is illustrated in Figure 8B. In addition, the reproducibility test results were also statistically analyzed with Grubbs’ and Dixon’s tests. The relative standard deviations of reproducibility test results and the *p* values of Grubbs’ and Dixon’s tests are summarized in Table S2B. The *p* values were lower than critical values, and accordingly, these data showed the acceptable reproducibility of the fabricated immunosensor.

Besides, the biosensor response to the 5 pg/mL antigen was assessed via Horwitz statistical analysis. The Horrat ratios of the repeatability and reproducibility tests were found to be 0.64 and 0.42, respectively, and these ratios were less than 2, which illustrated the acceptableness of the biosensor. Moreover, the sensing responses for the 5 pg/mL target antigen were investigated using the *T* and *F* tests. The *t* test compared the means of two test results, and the calculated *T* value (0.20) was smaller than the *T*-critical two-tail value (2.10). This result displayed that there was nothing noteworthy. The distribution of biosensor response results was also examined with the *F* test. The *F* value of the data set (2.284) was lower than the critical *F* value (4.026), and it indicated the sample averages were not considerably different from each other. Figure 8C illustrates the quality control diagram of the biosensor. The reference, upper and lower control, and upper and lower warning values were 5, 5.278 and 4.722, and 5.185 and 4.815 pg/mL, respectively. Consequently, this sensing system had an admissible analytical performance for resistin analysis.

**Figure 9:**
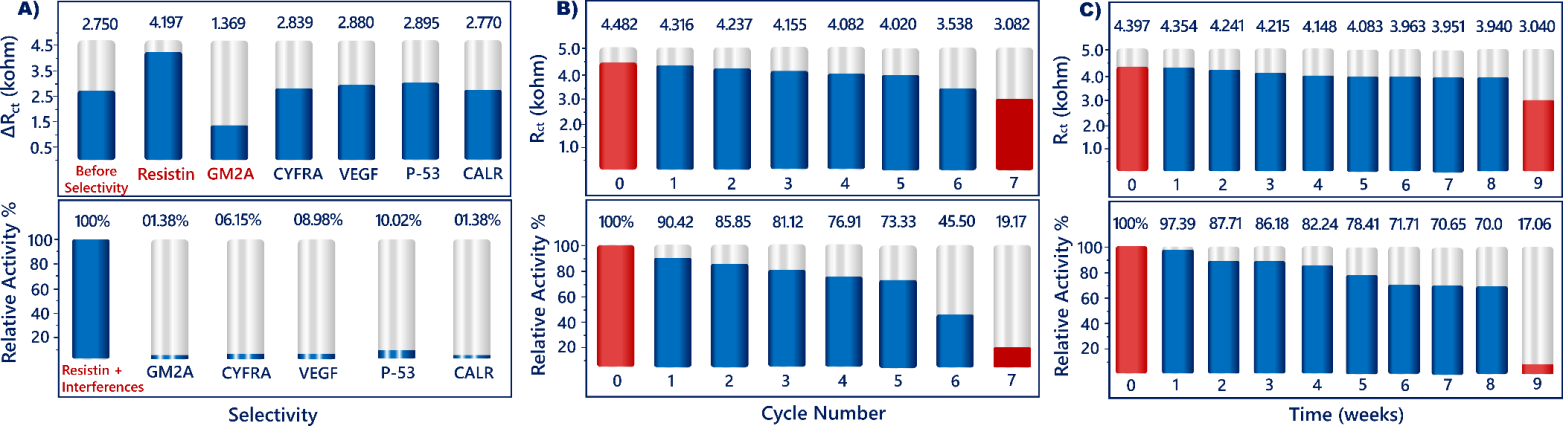
Evaluation of the selectivity (A), reusability (B), and long-term storage (C) of the resistin biosensor.

The selectivity of the immunosensor was analyzed in the presence of different biomarkers (NSE, VEGF, GM2A, CYFRA 21-1, and CALR) in phosphate buffer. When the resistin biomarker was added to the prepared phosphate buffer, it illustrated a high signal because of the specific interaction between anti-resistin and resistin. This change indicated the proposed immunosensor had good selectivity for resistin determination (Figure 9A). In addition, the designed BSA modified electrodes were introduced at three different concentrations of resistin and other biomarkers (0.1, 5, and 10 pg/mL). As seen in Figure S5, these interference biomolecules caused very weak impedimetric responses.

Regeneration is known as a process that overcomes the attractive forces between the bio-receptor and target analyte, and this process can substantially enhance the practicability of the biosensor. In different studies, to regenerate the electrode surface, high- or low-pH buffers have been applied to the electrodes. The variation in pH changes the enthalpic state and the relative charges between the bio-receptor and the target analyte. During the variations, the side groups of proteins can be ionized, or the ionic strength of the environment around the biomolecule can be changed. In order to overcome the attractive forces between the anti-resistin and resistin antigen, 0.01 M HCl was utilized in this study. The ITO/*P(TdiEpx)*/anti-resistin/BSA/resistin electrodes were treated with this acidic solution for 4 min. The EIS response of the bioelectrode was measured after regeneration cycles and resistin immobilization. After five cycles, the EIS signals reduced to 73.33% of the initial response, indicating the biosensor had excellent regeneration ability (Figure 9B).

For the storage stability test, the ITO/*P(TdiEpx)*/anti-resistin/BSA bioelectrodes were stored at 4 °C and utilized for the resistin analysis at intervals of 1 week within 10 weeks. The obtained results illustrated that the bioelectrode response diminished to 70.06% of the initial response after 8 weeks, indicating the suggested sensor had acceptable storage stability (Figure 9C).

### 3.4 Application of This Immunosensor in Biological Samples

**Figure 10:**
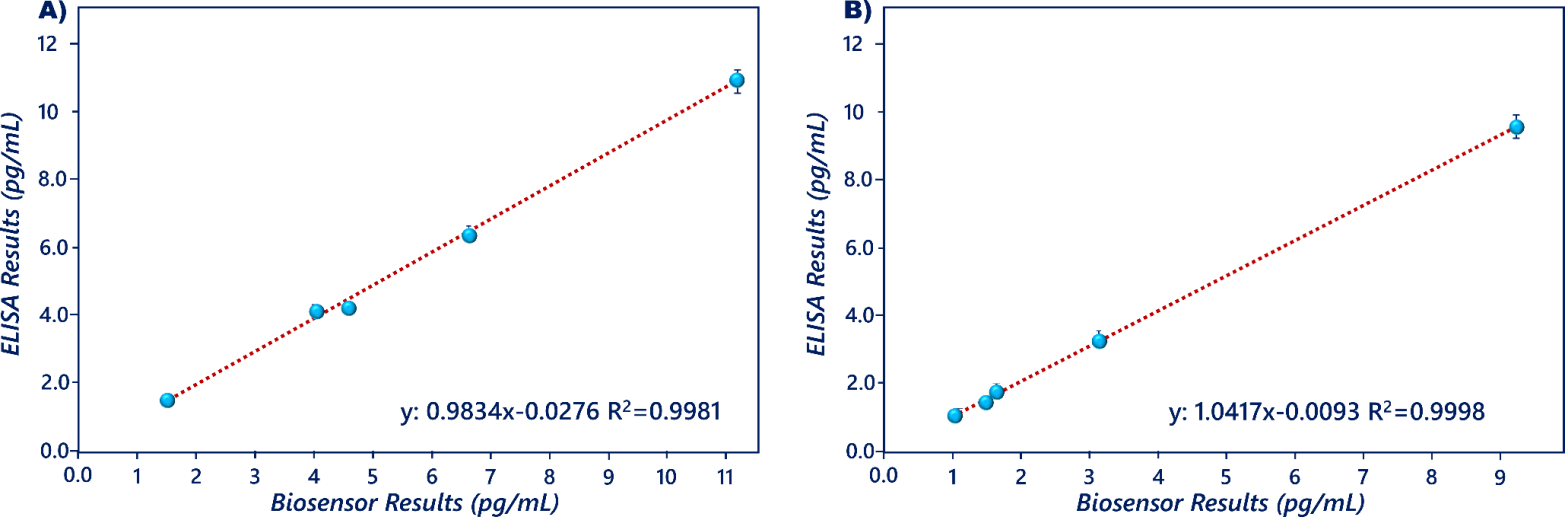
Correlation plots drawn by using the human serum (A) and saliva (B) results of ELISA kit and proposed biosensor.

To illustrate the possibility of implementing the immunosensing system for clinical diagnostics, the resistin level of serum and saliva samples was analyzed with this system. Before the measurements, serum and saliva samples were diluted 20 and 50 times with phosphate buffer, respectively. The serum and saliva results were compared to the results of the resistin ELISA kit. The results of the quantification coefficient and slope of human serum and saliva samples were 0.9981–0.9834 and 0.9998–1.0417, respectively (Figure 10A,B). In conclusion, a good correlation between immunosensor and ELISA was obtained, and the suggested system could be successively applied to the samples.

**Figure 11:**
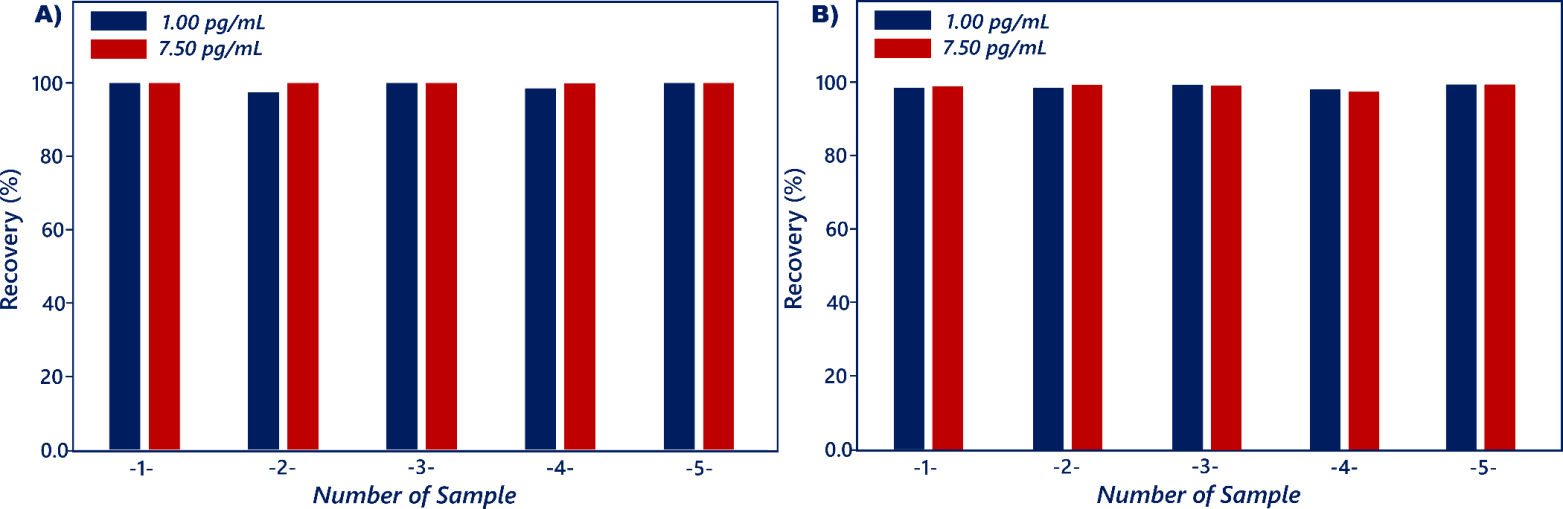
Recovery rates obtained from serum (A) and saliva (B) samples.

**Figure 12:**
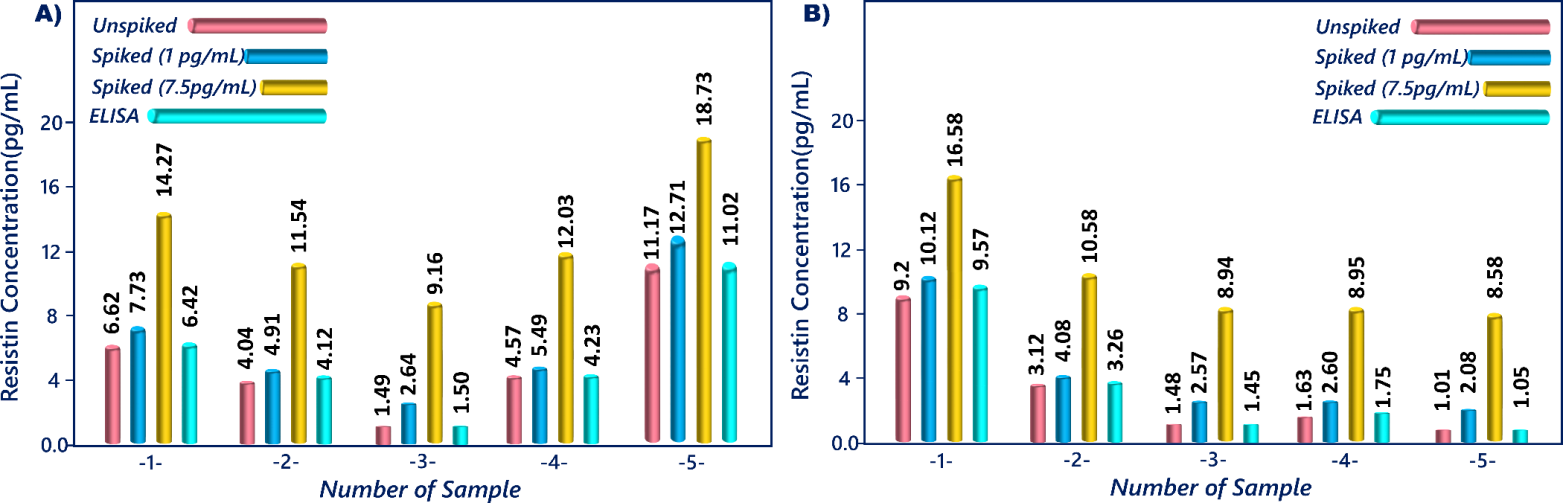
Bare diagram of serum (A) and saliva (B) results obtained before and after standard addition technique.

With the aim of evaluating the reliability of the suggested sensing system, different amounts of resistin (1 and 7.5 pg/mL) were spiked into the samples. To diminish the effect of matrix on the analysis results, the serum and saliva samples were diluted 20- and 50-fold in phosphate buffer, respectively. The concentrations of resistin in actual samples were measured by utilizing the fabricated immunosensor. As viewed in Table 1B,C, the detected amounts of resistin were highly similar to the spiked ones, and recoveries in serum and saliva samples were found in the ranges of 97.26–106.29% and 95.81–106.97%, respectively (Figure 11A,B). In addition, the bare diagrams of recycling test results for serum and saliva samples are illustrated in Figure 12A,B, respectively. The recycling test results displayed that the constructed sensor could be used to determine resistin in these samples for the early diagnosis of obesity.

## 4 Conclusion

A new efficient platform based on a conjugated *P(TdiEpx)* polymer was constructed for the development of electrochemical resistin immunosensor. The synthesized polymer interface had good conductivity and a large surface area for anti-resistin binding. After electropolymerization of *TdiEpx* on the disposable ITO electrode surface, attachment of anti-resistin was achieved via covalent bonds formed between the epoxy groups of *P(TdiEpx)* and amino groups of anti-resistin. The successful fabrication of the immunosensor was proven through EIS and CV analyses. The constructed immunosensor illustrated impedimetric signals with a proportionally increasing over a resistin concentration range of 0.0125–15 pg/mL. The proposed immunosensor showed excellent parameters with 4.17 fg/mL LOD, 16.87 fg/mL LOQ, and 1.378 kohm pg mL^–1^ cm^2^ sensitivity. Further, the immunosensor’s performance in terms of storage stability, selectivity, repeatability, reproducibility, and reusability were analyzed. Additionally, the suggested immunosensing system and ELISA were utilized for human serum and saliva sample analyses. Both measurements of resistin in clinical samples illustrated good correlations (serum, *R*^2^ = 0.9981; saliva, *R*^2^ = 0.9998). Compared to the ELISA method, this immunosensor had a high analytical sensitivity, even at low levels of resistin. Consequently, the proposed design of the immunosensor utilized in this study may be potentially applied for the determination of several other biomolecules in clinical diagnosis without the requirement for any sample pretreatment.

## Supporting Information Available

Chemical characteristics of monomer, *R*_ct_ values of EIS spectra, and RSDs of repeatability and reproducibility studies (PDF)
