# Supplementary material for: Label-Free Electrochemical Immunosensor Based on Conjugated Polymer Film Coated Disposable Electrode for Ultrasensitive Determination of Resistin Potential Obesity Biomarker
Source: ACS Appl Bio Mater. 2024 Feb 23;7(3):1820–30. doi: 10.1021/acsabm.3c01231 (PMC10952011; doi:10.1021/acsabm.3c01231)
Supplement: Supplementary file 1 — mt3c01231_si_001.pdf [file mt3c01231_si_001.pdf]

Electronic Supplementary Information

Label-free electrochemical immunosensor based on conjugated polymer film coated disposable electrode for ultrasensitive determination of resistin potential obesity biomarker

Elif Burcu Aydın[a*], Muhammet Aydın[a], Mustafa Kemal Sezgintürk[b]

[a]Tekirdağ Namık Kemal University, Scientific and Technological Research Center, Tekirdağ-Turkey
[b]Çanakkale Onsekiz Mart University, Faculty of Engineering, Bioengineering Department, Çanakkale, Turkey

***Corresponding Author:** Assoc. Prof. Elif Burcu Aydın
e-mail: ebbahadir@nku.edu.tr

Tel:+90 282 250 11 37

1. Chemical Characterization of Monomer

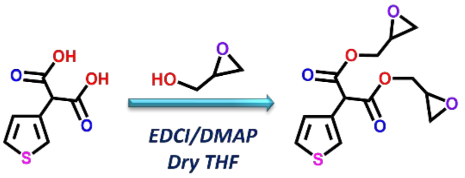

Fig. S1. Synthesis pathways of Double epoxy functional group-substituted thiophene monomer (*TdiEpx*).

Double epoxy functional group-substituted thiophene monomer (*TdiEpx*), a colorless liquid, was synthesized from a simple esterification reaction between 3-thiophenemalonic acid and glycidol at room temperature under argon flux by minor modification of the processes present in the Steglich esterification method. The process of synthesis routes of monomer (*TdiEpx*) is displayed in figure S1. The chemical structures of monomer were examined using FTIR, Raman, and ^{1}H NMR spectroscopy to show the effectiveness of the synthesis methods and to ascertain their chemical compositions.

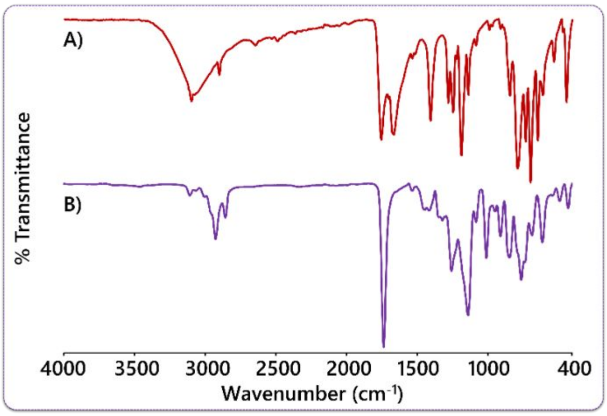

Fig. S2. The FTIR spectra of A) 3-thiophenemalonic acid and B) thiophene diepoxy monomer (*TdiEpx*).

The FTIR spectra of 3-thiophenemalonic acid and thiophene diepoxy monomer (*TdiEpx*) are given in Fig. SI-2. The 3-thiophenemalonic acid showed a broad peak around 3500-3000 cm^{-1}, originating from the -OH end groups, which disappeared completely after the reaction with glycidol (Fig.S2A). The successful synthesis of monomer (*TdiEpx*) was evidenced by the presence of a new carbonyl stretching peak at 1738 cm^{-1}, which was attributed to C=O stretching vibration of ester groups in thiophene monomer (Fig. S2B) [1-2]. The aromatic and aliphatic υ(C-H) stretching peaks of thiophene ring were seen

at 3150-3050 cm^{-1} and 3000-2850 cm^{-1}, respectively [3]. Furthermore, the characteristic signals of epoxy groups in monomer were seen at 908 and 844 cm^{-1} [4-6]. The two bands observed around 1137 cm^{-1} and 685 cm^{-1} were attributed C-S-C asymmetric and symmetric stretching vibrations in thiophene ring [7-8]. The chemical structures of 3-thiophenemalonic acid and thiophene diepoxy monomer (*TdiEpx*) were also examined via Raman spectral technique. Raman spectroscopy is more commonly used spectral technique which supports to FTIR spectral results. The Raman spectra of the monomers are given in Figure S3. The carbonyl stretching of thiophene diepoxy monomer in the Raman spectra was seen at 1738 cm^{-1}. The characteristic signals of epoxy group in monomer were seen at 925 cm^{-1} and 861 cm^{-1} and confirmed the presence of epoxy ring in monomer [9-10].

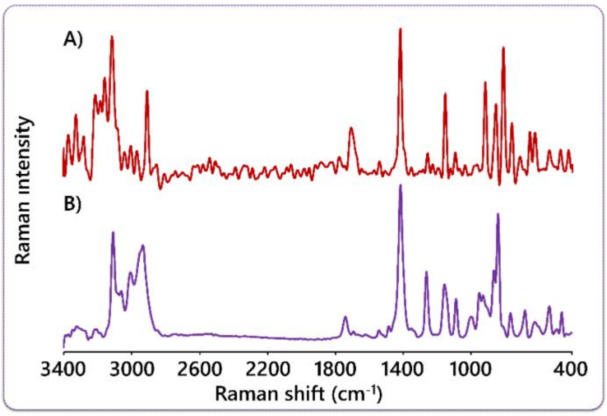

Fig. S3. The Raman spectra of A) 3-thiophenemalonic acid and B) thiophene diepoxy monomer (*TdiEpx*).

The chemical structure of thiophene monomer (*TdiEpx*) was determined by proton NMR spectroscopy and it was dissolved in deuterated chloroform ($CDCl_3$). The three peaks in thiophene rings appeared in the aromatic region at 7.30 ppm (H_a), 7.05 ppm (H_b) and 7.17 ppm (H_c) which were with the integration ratios 1:1:1. The proton (H_d) of thiophene side group was seen at 5.30 ppm. The peaks at 4.48 and 3.95 ppm ($H_{e1,2}$) were attributable to the methylene group on monomer side group. The protons of oxirane ring were seen at 3.23 ppm (H_f), 2.85 and 2.63 ppm (H_g). The integration ratios of Ha:Hb:Hc:Hd:He:Hf:Hg was calculated as 1:1:1:1:2:1:2.

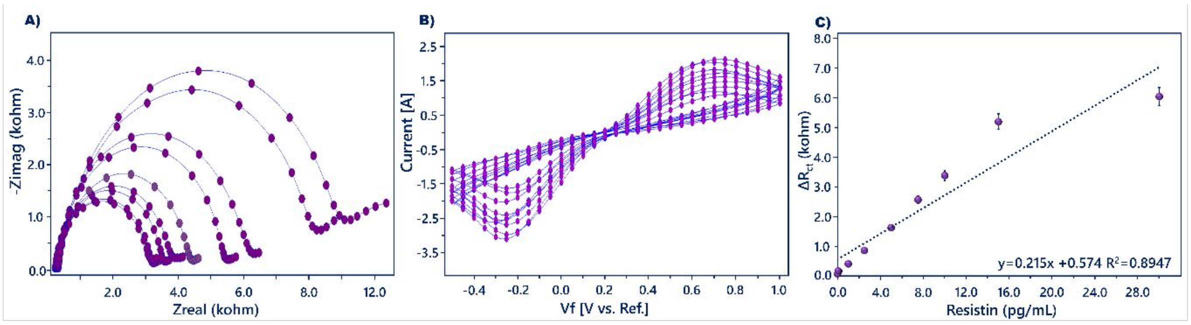

Fig. S4. The response of the biosensor to 30 pg/mL resistin concentration; EIS (A), CV (B), and calibration curve (C).

Table S1. The fitted EIS spectra result of electrode modification steps (A) and resistin antigen immobilized electrodes (B).

**(A) Biosensor Surface**	**R_{ct} (kohm)**
ITO-TdiEpx	2.045
ITO- TdiEpx/anti-resistin	2.418
ITO- TdiEpx/anti-resistin/BSA	2.683
ITO- TdiEpx/anti-resistin/BSA/resistin	3.562

**(B) Biosensor Surface**	**R_{ct} (kohm)**
ITO- TdiEpx/anti-resistin/BSA/resistin (0.0125pg/mL)	2.801
ITO- TdiEpx/anti-resistin/BSA/resistin (0.1 pg/mL)	2.867
ITO- TdiEpx/anti-resistin/BSA/resistin (1 pg/mL)	3.112
ITO- TdiEpx/anti-resistin/BSA/resistin (2.5 pg/mL)	3.562
ITO- TdiEpx/anti-resistin/BSA/resistin (5 pg/mL)	4.330
ITO- TdiEpx/anti-resistin/BSA/resistin (7.5 pg/mL)	5.266
ITO- TdiEpx/anti-resistin/BSA/resistin (10 pg/mL)	6.081
ITO- TdiEpx/anti-resistin/BSA/resistin (15 pg/mL)	7.895

Table S2. p-values of repeatability (A) and reproducibility (B).

**A)**	**0.1 pg/mL**	**10 pg/mL**	**37.5 pg/mL**
RSD	6.27	2.85	2.20
p-value of low Grubbs' test	1.855	1.384	1.755
p-value of high Grubbs' test	1.351	1.519	1.508
p-value of low Dixon's test	0.25	0.062	0.137
p-value of high Dixon's test	0.062	0.185	0.206
**B)**	**0.1 pg/mL**	**10 pg/mL**	**37.5 pg/mL**
RSD	6.30	1.88	3.40
p-value of low Grubbs' test	2.029	2.011	1.609
p-value of high Grubbs' test	1.302	1.159	1.029
p-value of low Dixon's test	0.364	0.333	0.004
p-value of high Dixon's test	0.000	0.000	0.067

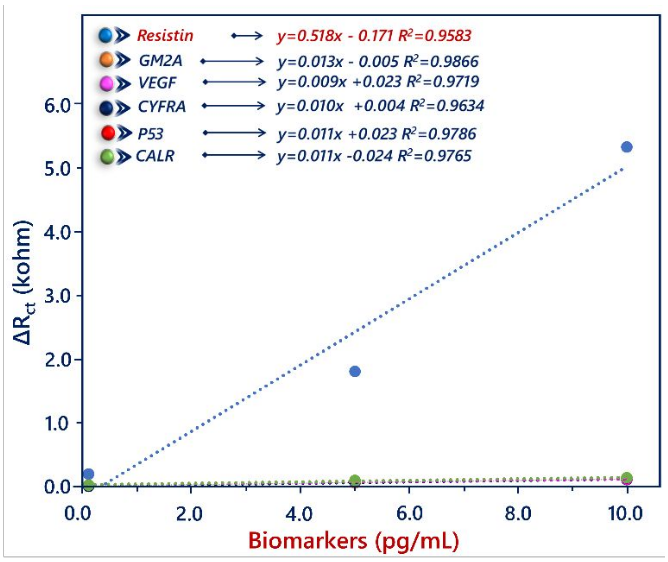

Fig. S5. The biosensor response to resistin and other biomarkers.

References

1. Wang, M.; Baek, P.; Voorhaar, L.; Chan, E. W. C.; Nelson, A.; Barker, D.; Travas-Sejdic, J., Long side-chain grafting imparts intrinsic adhesiveness to poly (thiophene phenylene) conjugated polymer. *Eur. Poly. J.* **2018,** *109*, 237-247.
2. Aydın, E. B.; Aydın, M.; Sezgintürk, M. K., Novel electrochemical biosensing platform based on conductive multilayer for sensitive and selective detection of CYFRA 21-1. *Sens. Actuators, B,* **2023,** *378*, 133208.
3. Foo, K.-L.; Ha, S.-T.; Yeap, G.; Lee, S., Mesomorphic behaviors of a series of heterocyclic thiophene-imine-ester-based liquid crystals. *Phase Transitions* **2018,** *91* (5), 509-520.
4. Edmondson, S.; Huck, W. T., Controlled growth and subsequent chemical modification of poly (glycidyl methacrylate) brushes on silicon wafers. *J.Mater. Chem.* **2004,** *14* (4), 730-734.
5. Canamero, P. F.; de la Fuente, J. L.; Madruga, E. L.; Fernández-García, M., Atom transfer radical polymerization of glycidyl methacrylate: a functional monomer. *Macromol. Chem. Phys.* **2004,** *205* (16), 2221-2228.
6. Jonsson, M.; Nyström, D.; Nordin, O.; Malmström, E., Surface modification of thermally expandable microspheres by grafting poly (glycidyl methacrylate) using ARGET ATRP. *Eur. Poly. J.* **2009,** *45* (8), 2374-2382.
7. Das, D.; Sen, K.; Maity, S., Studies on electro-conductive fabrics prepared by in situ chemical polymerization of mixtures of pyrrole and thiophene onto polyester. *Fibers Polym.* **2013,** *14*, 345-351.
8. Sahin, E.; Camurlu, P.; Toppare, L.; Mercore, V. M.; Cianga, I.; Yagˇcı, Y., Conducting copolymers of thiophene functionalized polystyrenes with thiophene. *J. Electroanal. Chem.* **2005,** *579* (2), 189-197.
9. Krappitz, T.; Feibusch, P.; Aroonsirichock, C.; Hoven, V. P.; Theato, P., Synthesis of poly (glycidyl 2-ylidene-acetate) and functionalization by nucleophilic ring-opening reactions. *Macromolecules* **2017,** *50* (4), 1415-1421.
10. Schneider, B.; Doskočilová, D.; Štokr, J.; Tlusťáková, M.; Kalal, J., Structure of the product of polymerization of 2, 3-epoxypropyl methacrylate in the presence of porous glass. *Acta Polym.* **1979,** *30* (5), 283-289.
